# Supplementary material for: Skeletal muscle transcriptional dysregulation of genes involved in senescence is associated with prognosis in severe heart failure
Source: Commun Med (Lond). 2026 Jan 16;6:97. doi: 10.1038/s43856-025-01362-z (PMC12891519; doi:10.1038/s43856-025-01362-z)
Supplement: Supplementary file 2 — Supplementary Information [file 43856_2025_1362_MOESM2_ESM.pdf]

Supplementary Information

Skeletal muscle transcriptional dysregulation of genes involved in senescence is associated with prognosis in severe heart failure

Eric Rullman*, MD, PhD; Alen Lovric, Michael Melin, MD, PhD; Rodrigo Fernandez-Gonzalo, PhD; Thomas Gustafsson, MD, PhD

* Corresponding author (eric.rullman@ki.se)

Supplementary Table 1 | Baseline clinical characteristics of all patients with HFrEF admitted to the Cardiology Department at Karolinska University Hospital during the study period and heart failure patients enrolled in the study.

	All HFrEF patients (N=328)	Study Patients (N=58)	P-value
Baseline			
Age	70.5 [62.8, 78.0]	71.0 [63.3, 74.0]	0.646
Sex: Female	78 (23.8%)	11 (19.0%)	0.501
Male	250 (76.2%)	47 (81.0%)	
BMI (kg/m2)	26.5 [23.5, 30.2]	28.0 [25.0, 30.0]	0.390
Exercise			
Functional status (NYHA-class)			
III	311 (94.8%)	56 (96.6%)	0.751
IV	17 (5.2%)	2 (3.4%)	
Blood			
NT-proBNP (ng/l)	2400 [1090, 6080]	1990 [934, 4850]	0.018
eGFR (ml)	59.0 [42.0, 75.0]	61.2 [45.6, 81.1]	0.303
Echocardiography			
LVEF (%)			
Moderate (LVEF 30-39%)	126 (38.4%)	10 (17.2%)	0.002
Severe (LVEF < 30%)	202 (61.6%)	47 (81.0%)	
Comorbidities			
Hypertension	167 (50.9%)	34 (58.6%)	0.474
Atrial fibrillation	167 (50.9%)	31 (53.4%)	0.777
Diabetes	100 (30.5%)	25 (43.1%)	0.068
Pharmacological treatment			
RAAS-blockade	307 (93.6%)	54 (93.1%)	0.778
Betablockers	318 (97.0%)	55 (94.8%)	0.424
Mineral receptor antagonist	162 (49.4%)	37 (63.8%)	0.063
Device theraphy			
CRT	63 (19.2%)	21 (36.2%)	0.006
ICD	84 (25.6%)	36 (62.1%)	1.38×10^{-08}

A two-sided Welch's two-sample t-test was used for continuous variables and Fisher's exact test was used for categorical variables. Exact nominal *P*-values are reported in the table. Missing data - HFrEF patients: BMI (214; 65.2%), NT-proBNP (65; 19.8%), Hypertension (10; 3.0%), Atrial fibrillation (2; 0.6%), Diabetes (1; 0.3%), Mineral receptor antagonist (3; 0.9%); Study Patients: LVEF (1; 1.7%). Abbreviations: BMI, body mass index; NYHA, New York Heart Association; NT-proBNP, N-terminal pro-B-type natriuretic peptide; eGFR, estimated glomerular filtration rate; LVEF, left ventricular ejection fraction; RAAS, renin-angiotensin-aldosterone system; CRT, cardiac resynchronization therapy; ICD, implantable cardioverter-defibrillator.

Supplementary Table 2 | Baseline characteristics of the propensity-matched controls samples from GTEx cohort and heart failure patients enrolled in the study.

	Propensity - matched GTEx control (N=58)	Study Patients (N=58)	P-value
Baseline			
Sex: Male	47 (81.0%)	47 (81.0%)	1
Female	11 (19.0%)	11 (19.0%)	
Age	68.0 [64.3, 69.0]	71.0 [63.3, 74.0]	0.002
BMI	27.9 [26.0, 31.2]	28.0 [25.0, 30.0]	0.958
Comorbidities			
Diabetes	26 (44.8%)	25 (43.1%)	1
Hypertension	48 (42.8%)	34 (58.6%)	0.008

A two-sided Welch's two-sample t-test was used for continuous variables and Fisher's exact test was used for categorical variables. Exact nominal P-values are reported in the table. Abbreviations: BMI, body mass index.
