# Supplementary material for: Skeletal muscle transcriptional dysregulation of genes involved in senescence is associated with prognosis in severe heart failure
Source: Commun Med (Lond). 2026 Jan 16;6:97. doi: 10.1038/s43856-025-01362-z (PMC12891519; doi:10.1038/s43856-025-01362-z)
Supplement: Supplementary file 3 — Description of Additional Supplementary Files [file 43856_2025_1362_MOESM3_ESM.pdf]

Description of Additional Supplementary Files

File name: Supplementary Data 1

Description: Gene set collections used in over-representation analysis. These gene sets were created using differentially expressed transcripts reported in original publications focused on long term bed rest, supervised exercise intervention, cancer cachexia, and cellular senescence. Each gene-set is available as a separate sheet, containing genes annotated with at least one commonly used identifier together with the corresponding statistical information.

File name: Supplementary Data 2

Description: The provided information refers to both initial co-expression network (IN), containing 8867 transcripts within 19 communities, and final co-expression network (FN) obtained by merging highly similar gene clusters, resulting with 1481 transcripts across 14 communities. This includes (1) general information of the network communities, such as number of genes and associations with regards to well-known pathways and biological processes. As a part of general information, we also provide statistical validation of these communities using external datasets. (2) Detailed network analysis including community-level gene expression differences between heart failure patients and their respective controls, over-representation analysis with respect to long term bedrest, exercise intervention, cancer cachexia and cellular senescence. For each community we also provide results of correlation analysis to clinical characteristics and association to prognosis. (3) On the individual gene level, we provide information from UniProt and human protein atlas, results of differential expression analysis, and association with prognosis.

File name: Supplementary Data 3

Description: This file contains all the data needed to reproduce the co-expression network shown in Figure 3.

File name: Supplementary Data 4

Description: This file contains all the data needed to reproduce the Figure 4 and 6.

File name: Supplementary Data 5

Description: This file contains all the data needed to reproduce the Figure 5.

File name: Supplementary Data 6

Description: This file contains all the data needed to reproduce the Table 1 and Supplementary Table 1.

Supplementary Note involving GTEx data in Figure 5, Figure 7 and Supplementary Table 2: The GTEx data was obtained through controlled access and cannot be redistributed by the authors of this study. Researchers interested in reproducing the results presented in this study can request data access through dbGaP system (link). The code behind the relevant figures is available from the corresponding author upon request.
